# Gender-based differences in work styles and career satisfaction in the retina subspecialty in Japan

**DOI:** 10.1007/s10384-026-01348-x

**Published:** 2026-05-06

**Authors:** Maiko Maruyama-Inoue, Annabelle A. Okada, Miho Nozaki, Hiroko Terashima, Yoko Miura

**Affiliations:** 1 Department of Ophthalmology, Yokohama City University Medical Center, 4-57 Urafune-cho, Minami-ku, Yokohama, Kanagawa 232-0024 Japan; 2 Department of Ophthalmology, Kyorin University School of Medicine, Tokyo, Japan; 3 Department of Ophthalmology, Nagoya City University East Medical Center, Nagoya, Japan; 4 Division of Ophthalmology and Visual Science, Graduate School of Medical and Dental Sciences, Niigata University, Niigata, Japan; 5 Institute of Biomedical Optics, University of Luebeck, Luebeck, Germany; 6 Department of Ophthalmology, University of Luebeck, Luebeck, Germany

**Keywords:** Retina subspecialty, Gender, Work life balance, Career satisfaction

## Abstract

**Purpose:**

To investigate differences in work styles and career satisfaction between women and men physicians in the retina subspecialty in Japan using a questionnaire survey.

**Study design:**

Cross-sectional study.

**Methods:**

A web-based questionnaire was sent as an e-mail link to all 3,027 current members of the Japanese Retina and Vitreous Society in August 2023 with responses collected anonymously in September 2023. The questionnaire consisted of 4 main categories: baseline characteristics, job satisfaction, life events and career advancement, and suitability as a retina specialist and workplace gender equality.

**Results:**

Valid responses were received from 614 members, representing a response rate of 20.3%. One-fifth of men (19.0%) were “very satisfied” with their job, much higher than the same for women (7.5%) (*P* < 0.001). Furthermore, there was a significant difference between men and women who “strongly agreed” or “agreed” that life events were an impediment to one's career (44.3% vs 74.9%) (*P* < 0.001). There was also a significant difference between men and women who answered “strongly agree” when asked whether they felt they were suited to work in the retina subspecialty (31.0% vs 13.4%) (*P* < 0.001).

**Conclusions:**

Our study identified and quantified marked differences in work style and career satisfaction between women and men. Such differences highlight gender-based challenges in the retina subspecialty that could be improved in order to enhance career satisfaction and career longevity of women retina specialists in Japan.

## Introduction

In terms of gender disparity, Japanese women do well on issues that involve health indices, attainment of tertiary education and labor force participation compared to Group of Seven and Organisation for Economic Co-operation and Development averages [1]. However, this is in sharp contrast to large gender gaps in Japan for wages, pension income, unpaid care and housework, publicly-listed company board seats, and parliament seats [1]. Furthermore, gender disparity remains despite government legislation in the form of an Equal Employment Opportunity Act that first went into effect in 1985, which explicitly states that “In all work management, discrimination on the basis of sex is prohibited,” “In some special cases, women can be prioritized,” and “Disadvantageous treatment by reason of marriage, pregnancy, childbirth, etc. is prohibited” [2]. For example, in 2021, compared to the average wage of male workers in Japan set at an arbitrary level of 100, the average wage level of female workers was 75.2 [3]. Furthermore, according to 2023 data, the proportion of women professors in university faculties (presidents, vice presidents, and professors) was 17.8%, much lower than reported for academic leadership in the United States [4–6].

Meanwhile, greater numbers of women have entered the physician workforce throughout the world in recent years, and this trend has occurred in Japan as well [7]. Women comprised 40.2% of Japanese high school graduates starting their 6-year course of undergraduate medical school in 2023 compared to 32.9% in 2012 [8, 9]. Thus, gender equality has taken on new meaning as women doctors are increasingly being viewed as a major source of labor. Continuation of work as a physician involves different issues for women compared to men, often due to child/elderly care and other unpaid work that may vary depending on the cultural norms in each country. In Japan, additional factors include a rapidly aging and shrinking society. The overall population is projected to decline from 2020 to 2070 by 30% while the proportion of elderly 65 years or older (with accompanying age-related healthcare needs) is projected to increase from 28.6% to 38.7% [10]. Hence, greater participation of women in medicine has found two groups of supporters in Japan; the first being women physicians themselves who desire self-fulfillment with respect to their careers, and the second being medical care facilities who need the women physicians to continue working in the healthcare labor force. The purpose of this study was to document the current state of work styles and career satisfaction of women physicians versus men physicians within the Japanese retina subspecialty.

## Methods

### Questionnaire design

We performed a cross-sectional study to investigate differences in work styles and career satisfaction between women and men, using a web-based questionnaire consisting of 4 main categories: baseline characteristics concerning training, employment type and family (13 items), job satisfaction (1 general question and 1 multiple-item question), life events and career advancement (1 general question and 1 multiple-item question), and suitability as a retina specialist and workplace gender equality (1 general question and 1 multiple-item question) based in part on a EURETINA Women in Retina Survey in 2021 [11]. Our questionnaire was specifically developed with Japanese retina physicians in mind, and an English translation is shown in Table 1. Except for items regarding baseline education and employment characteristics, all questions were formatted such that the answers utilized a 5-point scale of, very satisfied, satisfied, neither, dissatisfied, very dissatisfied or, strongly agree, agree, disagree, strongly disagree, uncertain.

**Table 1.** Summary of questionnaire

Baseline Characteristics	1. Gender
	2. Age group by decade
	3. Employment type
	4. Employment status
	5. Experience of temporary leave of absence
	6. Doctoral degree
	7. Ophthalmology board certification
	8. Studied abroad for research
	9. Currently performing cataract surgery
	10. Currently performing vitreoretinal surgery
	11. Current marital status
	12. Job of spouse/partner (medical doctor or not)
	13. Children 18 years or younger
Job satisfaction	General question: "How satisfied are you with your current work?"
	Multiple item question: "Do the following statements apply to you?"
	1. I am confident in my role and in my abilities.
	2. I am given support for personal development and growth.
	3. I feel supported by my organization when I am dealing with personal and family issues.
	4. I do not feel sexism or gender stereotypes in the workplace.
	5. I am satisfied with my current salary.
	6. In general, I have a good work life balance.
Life events and career advancement	General question: "Do you think that life events hamper your career advancement?"
	Multiple item question: "What is the greatest barrier to career advancement?"
	1. Difficulty balancing life events with work
	2. Anxiety over my own health
	3. Low salary
	4. Overburdened with work
	5. Environment of persistent bias and gender stereotypes that favor men
	6. Workplace lack of understanding and flexibility when dealing with life events
	7. Absence of role models
	8. Despite my efforts, career advancement cannot be expected in my current environment.
Suitability as a retinal specialist and workplace gender equality	General question: "Do you think you are suited to work in the retina subspecialty?"
	Multiple item question: "What do you think is important to attain gender equality in the retina subspecialty?"
	1. Improvement of the surrounding environment (providing nursery and child care facilities, etc.)
	2. Improvement of workplace management for overnight call and caring for emergency patients
	3. Change in attitudes of both men and women regarding child/elderly care
	4. Receive fair consideration (salary) for work I do
	5. Workplace that allows promotion based fairly on degree of contribution
	6. Environment that promotes easy consultation, such as by a mentorship program
	7. Individualized system to support skill improvement, such as by a training program
	8. Set gender-based quotas for society board seats or conference speakers

### Survey implementation and collection

The questionnaire survey was conducted using Google Forms sent as an e-mail link to 3,027 current active members of the Japanese Retina and Vitreous Society (JRVS) in August 2023 with responses collected anonymously in September 2023. Membership in the JRVS is open to all Japanese ophthalmologists interested in clinical care and basic science research in the field of vitreoretinal diseases and, by report of the JRVS administrative office (email communication), was comprised of 2,225 men (73.5%) and 802 women (26.5%) for the year 2023. This research was conducted with the approval of the President and Executive Directors of the JRVS, and the results were presented in part at the Women in Japan Retina symposium, the first ever on this theme, on November 25, 2023 at the 62nd Annual Meeting of the JRVS held in Yokohama. Ethics review was deemed not necessary for this study by the ethics committee of Yokohama City University because it was a questionnaire survey intended for ophthalmologists. Of note, the JRVS does not have a society-based ethics committee and does not have an ethics approval mechanism. At the time of responding to the online survey, written informed consent was obtained from all participants for their voluntary participation in the research.

### Statistical analysis

Answers were collated in a contingency table, and Fisher’s exact test was used to compare answers to each question between men and women. All statistical analyses were performed using IBM SPSS Statistics for Windows (Version 28.0) (IBM Corp.), and *P* < 0.05 was considered statistically significant.

## Results

### Baseline characteristics

Of the 3,027 JRVS members to whom the questionnaire link was sent, valid responses were received from 614 members, representing a response rate of 20.3%. Of responders, 368 (59.9%) identified as men and 239 (38.9%) identified as women. In addition, 2 responders (0.3%) selected gender unspecified and 5 (0.8%) had no answer selected for gender. Data from the 607 responders who identified with a gender (368 men and 239 women) was analyzed for the study. This represented 16.5% of current JRVS members who are men versus 29.8% of members who are women, reflecting a statistically significant difference in responders by gender (*P* < 0.001).

Baseline characteristics by responder gender are shown in Table 2, including age grouped by decade, employment status, attainment of doctoral (Ph.D.) degree, ophthalmology board certification, experience studying abroad, and whether cataract and/or vitreoretinal surgery were currently being performed. There were no significant differences between men and women in terms of age or attainment of board certification. With respect to employment status, men were more likely to be university faculty members or doctors in private practice compared to women (*P* < 0.001). Furthermore, men were more likely to have obtained a doctoral degree, experienced studying abroad for research, currently performing cataract surgery or currently performing vitreoretinal surgery compared to women (*P* = 0.001, *P* < 0.001, *P* < 0.001, and *P* < 0.001, respectively). In contrast, women were more likely to be employed at reduced working hours or on a part-time basis compared to men (*P* < 0.001). In addition, women were significantly more likely to have had taken temporary leave of absence, be single after divorce or never married, have a spouse or partner who is a medical doctor, or have no children compared to men (*P* < 0.001, *P* < 0.001, *P* < 0.001, and *P* = 0.008, respectively).

**Table 2.** Baseline characteristics

		Men (n=368)		Women (n=239)		*P*-value
		n	%	n	%	
Age	Twenties	18	4.9	13	5.4	0.380
	Thirties	107	29.1	73	30.5	
	Forties	100	27.2	73	30.5	
	Fifties	105	28.5	51	21.3	
	Sixties and over	38	10.3	29	12.1	
Professional status	Faculty (professor, associate professor and senior lecturer)	104	28.3	37	15.5	<0.001
	Assistant professor, clinical fellow and resident	116	31.5	91	38.1	
	Employed doctor other than above	79	21.5	82	34.3	
	Doctor in private practice	60	16.3	21	8.8	
	Unemployed	0	0	3	1.3	
	Others	9	2.4	5	2.1	
Employment status	Full-time	337	91.6	168	70.3	<0.001
	Reduced working hours	8	2.2	35	14.6	
	Part-time	19	5.2	23	9.6	
	Not working	2	0.5	8	3.3	
	Others	2	0.5	5	2.1	
Have you had a temporary leave of absence?	Yes (less than one year)	15	4.1	98	41.0	<0.001
	Yes (more than one year, currently working)	5	1.4	37	15.5	
	Yes (more than one year, still not working)	1	0.3	4	1.7	
	No	347	94.3	100	41.8	
Do you have a doctoral degree?	Yes	226	61.4	115	48.1	0.001
	No	142	38.6	124	51.9	
Do you have a certified doctor?	Yes	316	85.9	212	88.7	0.326
	No	52	14.1	27	11.3	
Did you study abroad for the research?	Yes	128	34.8	34	14.2	<0.001
	No	240	65.2	205	85.8	
Do you perform cataract surgeries?	Yes	326	88.6	140	58.6	<0.001
	No	42	11.4	99	41.4	
Do you perform vitrectomy surgeries?	Yes	258	70.1	73	30.5	<0.001
	No	110	29.9	166	69.5	
Marital status	Married (including common-law marriages)	327	88.9	178	74.5	<0.001
	Single after divorced	6	1.6	20	8.4	
	Unmarried	35	9.5	41	17.2	
Jobs of the spouse or partner	Doctor	107	29.1	133	55.6	<0.001
	Non-doctor	229	62.2	53	22.2	
	No spouse or partner	32	8.7	53	22.2	
Do you have children under 18?	Yes (one)	80	21.7	49	20.5	0.008
	Yes (two)	117	31.8	57	23.8	
	Yes (three or more)	49	13.3	22	9.2	
	No	122	33.2	111	46.4	

### Job satisfaction

**Fig 1.**
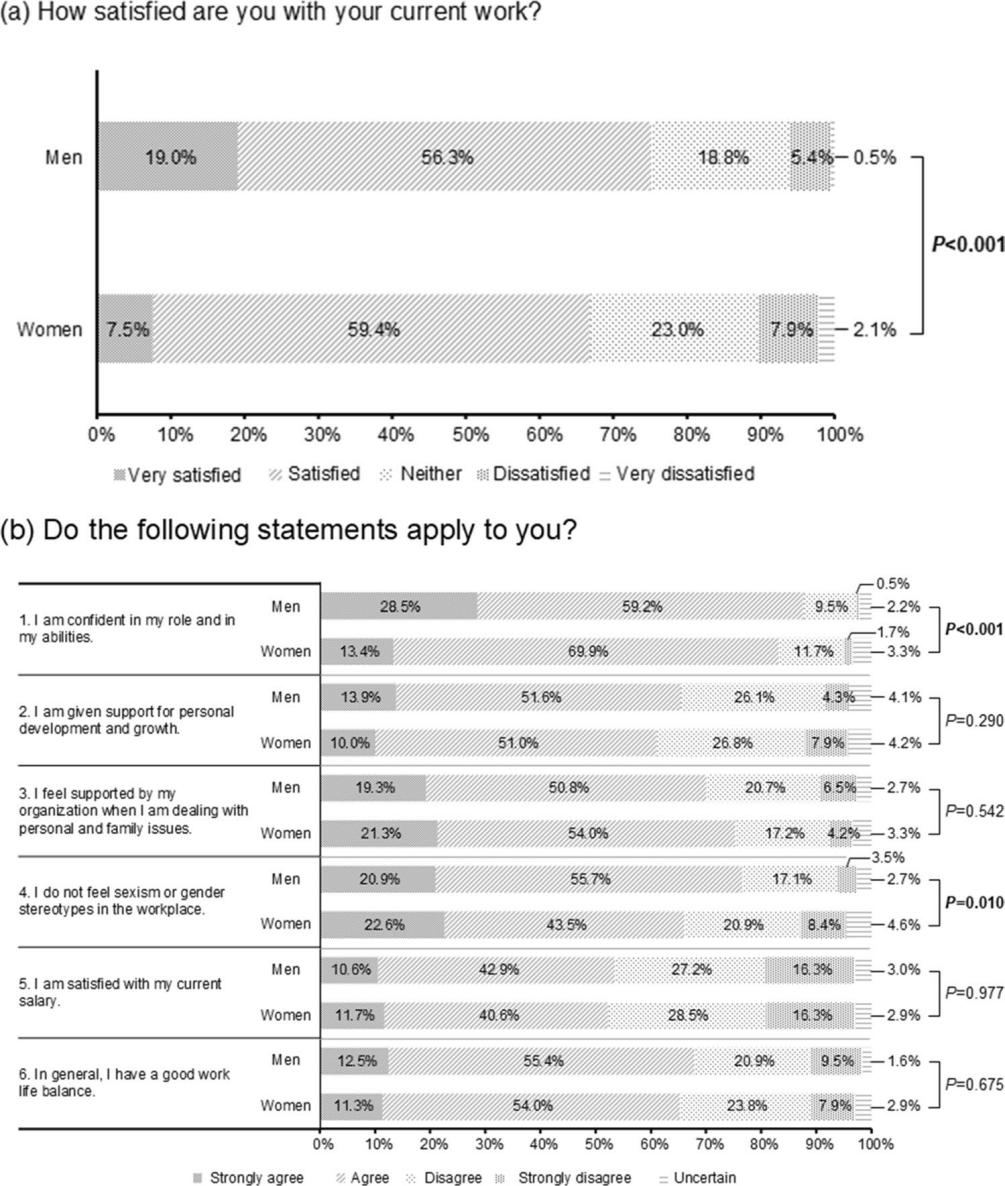
Questionnaire results by gender regarding job satisfaction. (a) Responses to the general question “How satisfied are you with your current work?” (b) Responses for the multiple-item question “Do the following statements apply to you?”

Overall, one-fifth of men (19.0%) were very satisfied with their job, much higher than the same for women (7.5%), while women had a higher proportion of being very dissatisfied or dissatisfied with their job (10.0%) compared to men (5.9%) (*P* < 0.001) (Fig. 1a). In addition, there was a significant difference between men and women in proportion who strongly agreed that they are confident in their role and in their abilities (28.5% vs 13.4%) (*P* < 0.001) (Fig. 1b). Furthermore, there was a disparity in the percentage between men and women in proportion of the sum of disagree and strongly disagree who had not felt any gender bias or discrimination in the workplace (20.6% vs 29.3%) (*P* = 0.010) (Fig. 1b).

### Life events and career advancement

**Fig 2.**
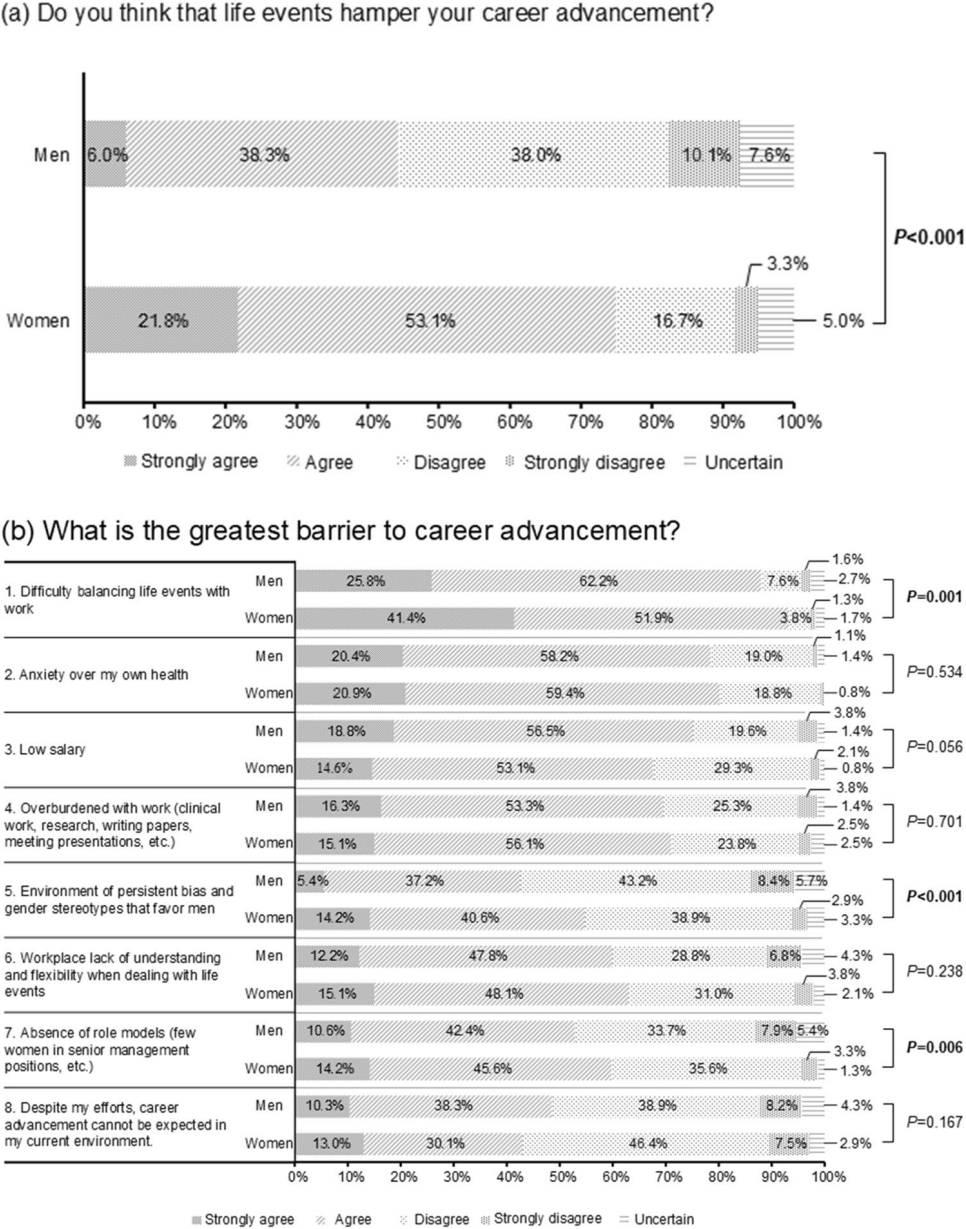
Questionnaire results by gender regarding life events and career advancement. (a) Responses to the general question “Do you think that life events (trying to get married, trying to get pregnant, childbirth, child rearing, divorce, elderly care, etc.) hamper your career advancement?” (b) Responses for the multiple-item question “What is the greatest barrier to career advancement?”

There was a significant difference between men and women who strongly agreed or agreed that life events were an impediment to one’s career (44.3% vs 74.9%) (*P* < 0.001) (Fig. 2a). Furthermore, 41.4% of women answered that they strongly agree when asked if they experienced difficulty in balancing life events and work, much greater than the 25.8% of men who answered similarly (*P* = 0.001) (Fig. 2b). When asked whether an environment of gender bias and gender stereotypes that favored men was an impediment to career advancement or not, women were more likely to answer strongly agree (14.2%) compared to men (5.4%) (*P* < 0.001) (Fig. 2b). Of note, majorities for both men (53.0%) and women (59.8%) answered strongly agree or agree that there was a lack of role models, and this difference was statistically significant (*P* = 0.006) (Fig. 2b).

### Suitability as a retinal specialist and workplace gender equality

**Fig 3.**
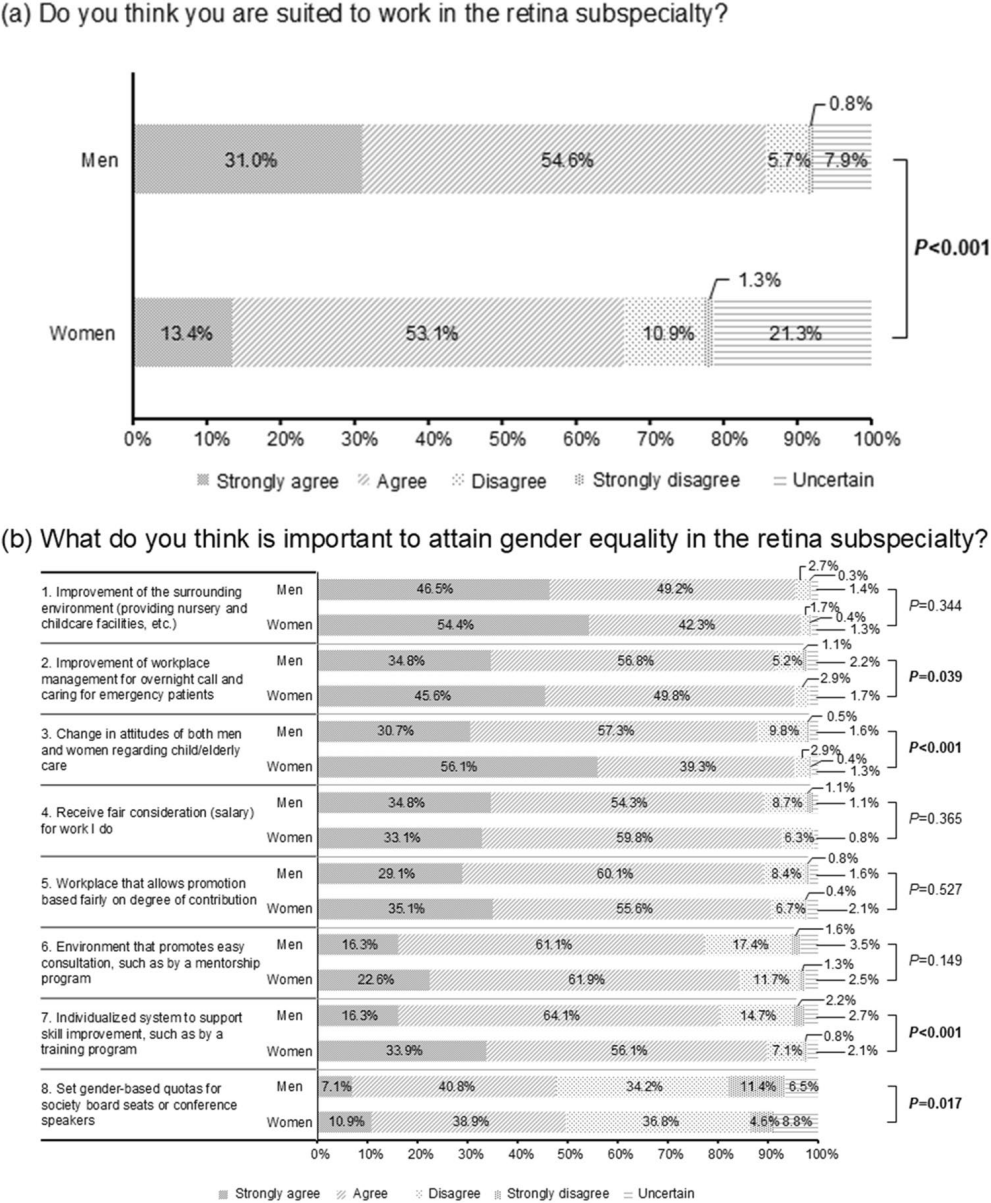
Questionnaire results by gender regarding suitability as a retina specialist and workplace gender equality. (a) Responses to the general question “Do you think you are suited to work in the retina subspecialty?” (b) Responses for the multiple-item question “What do you think is important to attain gender equality in the retina subspecialty?”

There was a significant difference between men and women who answered strongly agree when asked whether they felt they were suited to work in the retina subspecialty (31.0% vs 13.4%) (*P* < 0.001) (Fig. 3a). In addition, when asked what is necessary for men and women to attain equality in career advancement, a significantly higher percentage of women answered strongly agree regarding improving workplace management for overnight call and caring for emergency patients, change in attitudes of both men and women regarding child/elderly care, and individualized system to support skill improvement, such as by a training program (*P* = 0.039, *P* < 0.001, and *P* < 0.001, respectively) (Fig. 3b). On the other hand, although opinions were mixed for both men and women regarding setting gender-based quotas for society board seats and conference speakers, men were more likely to answer strongly disagree to such quotas compared to women (11.4% vs 4.6%) (*P* = 0.017) (Fig. 3b).

## Discussion

Since the proportion of women medical doctors in Japan is increasing [7–9], there are concerns that life events such as childbirth and child/elderly care will be major constraints for women to continue full-time work as physicians in the medical workforce. Indeed, in our study, women were more likely to report reduced working hours or part-time work compared to men, consistent with a previous study conducted by the Japanese Society of Cataract and Refractive Surgery [12]. This pattern of less than full-time working by women physicians is related to inherent Japanese society structural issues that regard housework and raising children as women’s responsibility and are not specific to the field of medicine. However, in the absence of resolving these structural issues, greater participation of women doctors in the medical workforce will be difficult. By conducting our questionnaire survey, we were able to objectively quantify to a certain degree the perceptions and experiences of women working specifically in the retina subspecialty. Our results may be summarized by the following 3 major points. First, women retina doctors have lower job satisfaction compared to men. Second, women doctors in the retina field feel difficulty in dealing with life events while working. Third, women feel that they are less suited to working in the retina subspecialty and, in order to enhance gender equality, believe that a change in the mindset of both men and women regarding child/elderly care is required.

In our study, although the proportion of women who answered very satisfied with their work was lower than men, the proportion who answered satisfied was almost the same. Figure 1b, question 1 also shows that the proportion of women who strongly agreed with being confident in their role and in their abilities was significantly lower than men. We suspect that one reason may be related to cultural norms which value women who are reserved and humble, perhaps leading women to underestimate their potential and feel less confident in the workplace. Indeed, a previous report from the U.S. describes women physicians rating their knowledge and skills in clinical research lower than the way men rated themselves [13]. Another study from the field of dermatology in Japan shows that women’s academic performance was self-reported to be lower than men at the level of assistant professor or lecturer [14]. Also, many women felt that persistence of gender stereotypes (unconscious bias) favoring men in the workplace played a large role in reducing perceived gender equality in our study (Fig. 1b, question 4) and in previous reports [15–17]. Although it may be difficult to improve the current situation immediately, educational programs at medical conferences, workshops for doctors, or university curriculum for medical students to learn about these biases, both conscious and unconscious, might lead to reduction in bias and enhancement of gender equality in the future.

Our survey also documented the difficulty women doctors face in balancing various life events (marriage, childbirth, child/elderly care, etc.) with work, and shows that women feel these events hamper advancement of their careers, a finding consistent with previous studies [18–20]. Such a result is expected, as it is deeply associated with the traditional division of labor in Japan in that women care for issues within the home while men work outside the home. Furthermore, women are also often required to take sudden unplanned leave to deal with unexpected childcare or eldercare needs, a role that society and family expect. When these emergencies arise, women physicians often need to request help from colleagues for coverage on short-notice. In the field of ophthalmology, we also suspect that at least a certain percentage of physicians, both men and women, have selected the specialty for the relatively few off-hour emergencies involved. This may be compounding the difficulty in finding ophthalmologists to fill-in for women who are suddenly unable to work due to unexpected family emergencies. One possible structural solution may be to establish a system of locum tenens whose work position is to fill in gaps that suddenly occur in the workforce. Another albeit weaker solution might involve financial or other incentives to colleagues at the same institution who are able and willing to step in and perform extra work to fill a gap in the workforce. However, as retina is highly specialized within ophthalmology, establishing a pool of locum tenens would not be easy, nor would it be easy to motivate full-time colleagues who may already feel overworked to perform additional work. Of note, monetary incentives have not been found to be particularly effective in preventing physician burnout [21].

Regardless, the need to support women working as medical doctors will only become greater in the coming years, as it is apparent that the proportion of incoming medical students who are women continues to rise in Japan [8, 9]. Therefore, it is essential to go further than merely identifying work life balance issues of and sympathizing with women doctors. Actual investment is required. A structural system needs to be built for the entire medical field that would incorporate new budgets, new management and training initiatives, and new personnel for the specific purpose of maintaining the ability for women physicians to continue working throughout their professional life. In addition, our questionnaire survey identified the lack of role models being one perceived barrier to career advancement for women doctors (Fig. 2b, question 7). Furthermore, since ophthalmology involves relatively less systemic management of patients compared to other specialties, individuals who originally value work-life balance may by choosing ophthalmology to begin with, leading to a self-selected group of biased physicians. Thus, there may already exist a strong unconscious bias on the part of the women in ophthalmology that child-rearing is the responsibility of women. This may in turn be causing a lower number of role models in full-time retina careers. Supporting diversity in top positions within universities and academic societies, as well as creating mentorship programs may help to alleviate this lack of role models.

The proportion of women who felt suited to the retina subspecialty tended to be lower than men. Unlike subspecialties that focus primarily on elective procedures, the retina subspecialty is characterized by a high volume of emergency surgery and time-sensitive outpatient treatments, such as intravitreal injections for chronic retinal diseases. These professional requirements often lead to unpredictable working hours and a heavier physical burden, which may disproportionately affect female physicians during major life events. Our findings highlight that these field-specific demands are significant factors contributing to the gender gap in career satisfaction and advancement within the Japanese retina community. Changing spousal expectations regarding child and elderly care, and increased support from colleagues for women with family caregiving responsibilities, may allow greater numbers of women doctors to continue working in the retina field.

Finally, regarding the introduction of quotas, although a greater proportion of men strongly opposed the idea compared to women, opinions were almost evenly divided overall for both men and women. In the German business world, the term “Thomas Kreislauf (in English, “Thomas cycle")” is used to describe the phenomenon whereby companies or organizations tend to select new executive board members, overwhelmingly men, who resemble their predecessors, reflecting a self-perpetuating pattern of homogeneity. The term draws on the fact that Thomas is a common male first name in Germany and is used symbolically to illustrate this phenomenon. Naturally, in line with this self-perpetuating dynamic, companies place a high priority on stability and thus tend to select what is familiar or adhere to selection criteria with known outcomes, resulting in low representation of women and minorities in executive positions [22]. Other unintended consequences include the suppression of active discussion, and reduced ability to keep up with rapid changes both internal and external to the company. The “Thomas Kreislauf” may also explain one aspect that women physicians face in terms of promotion and/or representation at the executive level within their organizations. This reduced percentage of women in leadership positions is observed in science professions in general. While the number of women receiving postgraduate degrees has been increasing, this has not led to a corresponding increase in women in faculty positions in science, technology, engineering and mathematics, the so-called “STEM” fields [23]. The establishment of quotas may be useful in temporarily breaking the “Thomas Kreislauf” and increasing representation of women doctors in leadership positions, with the ultimate goal of increasing career satisfaction and prolonging medical careers. Actually, our organization, JRVS, began implementing a quota system in 2025 requiring that women constitute 20% (8/40) of the board members, including President, Executive Directors and Directors [24].

The limitations of our study include the overall low response rate of 20.3% of JRVS members. In addition, there was a significant difference in response rate between men and women members, perhaps due to relatively dissatisfied members being more likely to respond to the survey. We acknowledge that this absence of formal validation may have led to a certain level of bias in our results. Furthermore, our current analysis did not stratify opinions by age or by employment type (e.g. university faculty member v. private practice), and one can easily imagine that the age of the responder, in particular, would have a large influence on their attitude towards gender issues. Also, although both medical and surgical retina fall under the broader category of retinal subspecialties, there are substantial differences in clinical practice and career trajectories between these two areas. Although data of responders were not collected in our study, results of questionnaire may be influenced by these differences. Additionally, we did not apply strict corrections for multiple comparisons in our statistical analysis. As this is an exploratory study aimed at identifying overall trends and generating initial hypotheses regarding gender disparities in the Japanese retina subspecialty, we prioritized the detection of potential disparities over conservative statistical adjustments. However, these findings should be validated in future confirmatory studies with larger cohorts.

In conclusion, our study identified and quantified marked differences in work style and career satisfaction between women and men. We believe that such differences highlight gender-based challenges in the retina subspecialty that could be improved in order to enhance career satisfaction and career longevity of women retina specialists in Japan.
